# Do Newspapers Foster Abortion?

**Published:** 1894-03

**Authors:** Henry Ryder-Taylor

**Affiliations:** Of the San Antonio (Tex.) Daily Light


For Texas Medical Journal.

### DO NEWSPAPERS FOSTER ABORTION?

BY HENRY RYDER-TAYLOR.
(Of the San Antonio (Tex.) Daily Light.)

FOR the November issue of the TEXAS MEDICAL JOURNAL I wrote an article, "Newspapers vs. the Doctors," and I was glad of the opportunity of placing the newspaper men's views before the profession, in order that we might better understand each other. For this purpose a fair showing was given; but in the same issue there are editorial comments that are inaccurate, or from which I differ, and which call for an answer.

In the first editorial comment, the newspapers, upon the authority of the *Lancet Clinic*, are accused of prostituting their telegraphic columns for medical advertising purposes, and it cites the alleged letter of George E. Gurrier to the *Anzeiger*, ordering the publication of two Amick advertisements as fake telegrams, and says that the same orders were sent to three hundred daily papers. My professional experience taught me that this, to say the least, was irregular, and I then doubted the accuracy of the statement, but I was not in a position to deny or affirm it. Since then I have written Mr. George E. Gurrier, who is a well known telegraphic correspondent of New York, and he gives an absolute denial to the statement. He further hints that the *Lancet Clinic* will have to answer for libel, and he gives reasons for the action that I am not at liberty to state. If Mr. Gurrier's statement is true, then this editorial comment, based upon false premises, is wrong, and the newspapers are vindicated of the charge it makes against them.

The second editorial comment makes a far more serious charge. It accuses the newspaper of fostering abortion! And why? Simply because it advertises preparations that, as I am informed, can and are legitimately used for the cure of female diseases, but which, if used in an unproper way, may and will produce abortion. This argument seems to be as illogical as it is unjust. There are many preparations advertised in the JOURNAL that, improperly used, would have the same effect; there are others that, used in a nefarious way, would cause death, and yet I would never think of accusing it of fostering abortion and murder. Then again, the newspapers simply advertise. It is the druggist who handles, sells and derives his profit from the sale of these articles, and it is he who knows their nature and properties. Who, then, is the most culpable? The newspaper or the druggist? Why, the druggist, and yet your whole censure falls upon the newspaper man, I protest against this injustice, and, I think, upon reconsideration you will realize the justice of my protest.

The law has made stringent laws respecting abortion, and on the whole I am inclined to think that this is well; but while man is aggressive and perfidious, and woman weak and trusting, these things will continue, and illicit offsprings will be created. Knowing and seeing what I have in the world, I have often wondered whether the premature abortion of a *fœtus* would not be preferable for humanity at large than seeing respectable families disgraced, and the offspring either murdered *ex-utero* or cast upon the world as a waif, to grow up as an outcast and a criminal, and perpetuate his or her viciousness in their offspring, a viciousness that is not inherent in nature, but created by circumstances. But mind, I am not advocating any such theory, but simply expressing a doubt that has crossed my mind as it has, probably, many others.

That abortion is carried on in the United States, and to an alarming extent, is an admitted fact, even among the married, who have the least excuse for the crime; and the criminal records show that qualified doctors have figured largely in those annals. There is, too, every reason to believe that a great number exist undetected, and can be found in every city. Even in San Antonio application for such purpose has been made, the condition verified, and the demand refused, yet the patients have been relieved of their burden. How, none know otherwise than surmise, yet the end has been attained. I have every faith in the honor of the medical practitioner, as a rule, and I believe that few would commit such a crime and incur its risks, but cupidity, necessity and circumstances are powerful stimulants, and there are black sheep in the whitest of flocks. In my remarks I am simply stating cold facts based upon my personal knowledge, and I have no wish to offend or cast a slur upon any reputable physician, for I honor the profession; but here I would rise to remark, have you ever known a reputable newspaper man to be engaged in abortion, or to advocate the crime? I contend that in advertising these preparations that have a lawful, medical and beneficial use, the newspapers are not wrong, and that, if they are misused, they are no more to blame than the doctor would be if his patient took an overdose or fatal dose of medicine, contrary to instructions. In the abuse, not the use, lies the danger. After these arguments I do not think any can truly say that newspapers foster abortion.

